# Hypofractionated stereotactic radiotherapy using coplanar VMAT for single small brain metastasis: dosimetric analysis and clinical outcomes

**DOI:** 10.3389/fonc.2025.1428922

**Published:** 2025-02-27

**Authors:** Jialu Lai, An Li, Xianhu Zeng, Jia Liu, Lin Zhou

**Affiliations:** ^1^ Radiotherapy Physics & Technology Center, Cancer Center and State Key Laboratory of Biotherapy, West China Hospital, Sichuan University, Chengdu, Sichuan, China; ^2^ Department of Oncology, Chengdu First People’ Hospital, Chengdu, Sichuan, China; ^3^ Thoracic Oncology Ward, Cancer Center and State Key Laboratory of Biotherapy, West China Hospital, Sichuan University, Chengdu, China

**Keywords:** hypofractionated stereotactic radiotherapy, small brain metastases, planning target volume margin, dosimetric parameters, clinical outcome

## Abstract

**Purpose:**

To evaluate the dosimetric parameters and clinical outcomes of hypofractionated stereotactic radiotherapy (HSRT) for small brain metastases [BMs; planning target volume (PTV) ≤ 4 cm^3^) via coplanar volumetric modulated arc therapy (C-VMAT).

**Methods:**

Between March 2019 and February 2023, 68 patients with a single BM treated with Linac-based HSRT (24–39 Gy in three fractions) via C-VMAT and a 3-mm PTV margin were enrolled in this retrospective analysis. A frameless head–neck–shoulder thermoplastic mask, whose immobilization accuracy is inferior to that of specialized mask fixation systems, was used to immobilize patients. Dosimetric parameters and clinical outcomes were evaluated.

**Results:**

C-VMAT provided clinically satisfactory treatment plans, with median gradient index, conformity index, homogeneity index, and PTV coverage values of 4.30, 1.05, 1.28, and 98%, respectively. The median volumes of normal brain tissue receiving 18 Gy, 21 Gy, and 23 Gy were 7.29 cm^3^, 5.33 cm^3^, and 4.40 cm^3^, respectively. High delivery accuracy was observed, with a gamma passing rate ≥90% for all plans. As of June 2023, the median follow-up time was 9.1 months. The intracranial objective response rate and disease control rate were 64% and 96%, respectively. The median intracranial progression-free survival was 26.9 (95% CI, 12.7–41.1) months. The 1- and 2-year local control (LC) rates were 91.5% (95% CI, 80.1%–100%) and 83.2% (95% CI, 64.6%–100%), respectively. The 1- and 2-year intracranial control rates were 70.9% (95% CI, 55.2%–86.6%) and 51.2% (95% CI, 32.6%–69.8%), respectively. Only four irradiated lesions progressed at the end of follow-up. The cerebral radiation necrosis rate of all patients was 7.4%.

**Conclusion:**

C-VMAT HSRT combined with a 3-mm PTV margin is an effective and safe treatment modality for small BMs.

## 1 Introduction

Stereotactic radiosurgery (SRS) is an advanced radiotherapy modality that delivers a highly precise radiation dose to a well-defined target volume, which achieves excellent local control (LC) while reducing the risk of neurocognitive deterioration compared with whole-brain radiotherapy (WBRT) for the treatment of brain metastases (BMs) (1, 2). Furthermore, numerous reports have indicated that hypofractionated stereotactic radiotherapy (HSRT) offers comparable efficacy to SRS while minimizing toxicity, especially for tumors located in or near organs at risk (OARs), such as the brainstem, cranial nerves, and cochlea (3, 4). Technical improvements, such as on-board image-guided radiation therapy (IGRT), high‐definition multileaf collimators (MLCs) and dedicated immobilization devices, have made non-invasive linear accelerator (Linac)-based volumetric modulated arc therapy (VMAT) a widely used delivery mode for BM HSRT (5–7).

Notably, the potential benefits of HSRT are suggested not only for large BMs but also for small BMs (8, 9). For instance, Faccenda et al. (9) found that Linac-based SRS treatments for small BMs with C-VMAT were feasible and resulted in encouraging clinical outcomes, comparable to those of other treatment approaches involving multiple non-coplanar arcs. However, in their study, the majority of patients (59 out of 70) received SRS (15–21 Gy in a single fraction) rather than HSRT. Moreover, VMAT, as an inverse planning technique, can achieve highly conformal dose distributions by simultaneously optimizing the MLC position, dose rate, and gantry rotation speed (10). However, excessive modulation can introduce dose calculation uncertainties, particularly when dealing with many small, irregular segments (11). This issue becomes especially pronounced in the treatment of very small BMs.

Specialized mask fixation systems, such as Qfix (12), are needed for SRS with a single fraction to ensure treatment accuracy. However, in our center, a conventional frameless head–neck–shoulder thermoplastic mask was used to immobilize patients for HSRT, and its immobilization accuracy is inferior to that of specialized mask fixation systems. Therefore, multifraction HSRT, typically with three fractions and an enlarged PTV border, was applied to overcome the deficiency of the fixation system in our center, and whether multifraction HSRT with C-VMAT and an enlarged PTV border can satisfy the clinical and dosimetric requirements is unknown. The present retrospective analysis aimed to evaluate the efficacy, toxicity, and dosimetric parameters of 3F-HSRT via C-VMAT for patients with small BMs with a 3-mm PTV margin who were unsuitable for or refused surgical resection and SRS.

## 2 Materials and methods

### 2.1 Patient population

This single-center, retrospective study included cancer patients with a single small BM (PTV ≤4 cm^3^) and a BM that received Linac-based C-VMAT 3F-HSRT via a 3-mm PTV margin. Patients were unsuitable for surgical resection and one-fraction SRS, which was evaluated by experienced neurosurgeons and radiation oncologists. Tumors located in or near (≤1 cm) critical normal tissues, such as the brainstem, optic nerves, cochlea, and capsula interna, were considered unsuitable for one-fraction SRS. Patients who refused surgical resection and one-fraction SRS were also enrolled. Patients were excluded if they received WBRT or SRS before HSRT. This retrospective study was conducted in accordance with the Declaration of Helsinki (as revised in 2013). This study was reviewed and approved by the ethics committee of West China Hospital, and the need to obtain individual consent for this retrospective analysis was waived.

### 2.2 CT/MRI simulation, target, and organs at risk delineation

All patients were immobilized in the supine position via a thermoplastic mask (Sichuan Ruidi Medical Science and Technology Co., Ltd., Chengdu, China). Magnetic resonance imaging (MRI) and contrast CT images with both 1-mm slice thicknesses were acquired. The GTV was defined as the contrast-enhanced region on the T1-weighted MRI. The PTV was generated by expanding the margin by 3 mm around the GTV in all dimensions. A 3-mm PTV margin is commonly used in our institution because the accuracy of thermoplastic mask immobilization is inferior to that of specialized mask fixation systems such as the Qfix Encompass thermoplastic mask (QFix Inc., Avondale, PA) (13, 14). OARs include the cochlea, optic chiasm/nerves, lenses, brainstem, basal ganglia, and eyeballs. Three-millimeter margins were around the lenses and brainstem to create the planning OAR volume (lenses PRV; brainstem PRV). The normal brain tissue (NBT) was equal to the brain minus the GTV. The median time from MRI localization to the start of treatment was 5 days (range, 3–8 days).

### 2.3 Treatment planning, dose specification, and delivery

Patients were treated with the C-VMAT delivery technique, which consisted of two coplanar arcs that rotated clockwise from 181° to 179° and counterclockwise from 179° to 181°. The planning isocenter was placed at the geometric center of the PTV. All treatment plans were designed utilizing 6 MV photon beams combined with flattening filter-free mode, which can achieve a maximum dose rate of 1,400 monitor units (MUs)/minute. All patients were treated at Edge^TM^ Linac (Varian Medical Systems, Palo Alto, CA), which was equipped with a 120-leaf high-definition MLC (5- and 2.5-mm leaf widths for the 28 outer and 32 central leaves, respectively).

Normal tissue objective parameters and five concentric ring structures were utilized in planning optimization to generate a steep dose fall-off and highly conformal target dose. The final dose was calculated via an anisotropic analytical algorithm (AAA) and a 1-mm grid size implemented in the Eclipse treatment planning system (v13.5; Varian Medical Systems, Palo Alto, CA). To improve delivery accuracy, a fixed-jaw technique was used for each plan with the jaw size fixed at 3 cm×3 cm. Each C-VMAT plan was normalized such that 100% of the prescription dose (P_D_) covered at least 95% of the PTV, with a maximum dose as large as 150% of the P_D_ accepted. The P_D_ was 24–39 Gy in three fractions, which were applied each other on a working day. The Varian OBI kV-CBCT system (Varian Medical Systems, Palo Alto, CA, USA) coupled with a robotic couch was utilized for patient alignment in six degrees of freedom (6DOF) prior to each treatment fraction. The setup process needed to be repeated until the tolerance was within 0.5 mm/0.5° in 6DOF.

### 2.4 Dosimetric parameters and delivery efficiency evaluation

For dosimetric analysis, parameters such as the PTV coverage, minimal dose received by 98% of the GTV (D_98%_), homogeneity index (HI), gradient index (GI), target conformity index (CI) and NBT sparing (the mean dose (D_mean_), absolute volume of NBT receiving ≥23 Gy (V_23Gy_), ≥21 Gy (V_21Gy_), and ≥18 Gy (V_18Gy_) were retrospectively collected on the basis of the clinical treatment plan. The CI (15) was defined as CI=V_100%_/V_PTV_, which indicates the ratio of 100% isodose volume (V_100%_) to the volume of the PTV (V_PTV_).

A CI value equal to 1 corresponds to ideal conformation. A CI greater than 1 indicates that the irradiated volume is greater than the target volume and includes NBT. If the CI is less than 1, the target volume is only partially irradiated (16). A particularly high CI value may increase the risk of cognitive impairment, radionecrosis, or other complications, while a particularly low CI value may cause a decrease in the LC rate. Usually, a CI value of less than 1.6 and greater than 0.95 was considered acceptable for treatment at our center. HI was defined as the ratio of the maximum dose to P_D_ (15). GI (17) was defined as the ratio of 50% isodose volume (V_50%_) to V_100%_. A lower value indicates a steeper dose fall-off outside the target and better sparing of the NBT. The beam-on time (BOT) and total number of MUs per fraction were used to evaluate the delivery efficiency.

A high-density diode array SRS MapCHECK^TM^ combined with a StereoPHAN^TM^ phantom (Sun Nuclear Corporation, Melbourne, FL, USA), which has been shown to be compliant with the recommendation of AAPM TG-101 (18), was used for treatment delivery accuracy evaluation. Gamma index analysis (19) was used to verify the C-VMAT delivery accuracy with three evaluation criteria (3%/1 mm, 2%/1 mm, and 2%/2 mm) with a 10% threshold.

### 2.5 Clinical efficacy and toxicity evaluation

Patients underwent brain gadolinium-enhanced MRI with 1.5-mm slice thicknesses before, 1 month after, and then every 2–3 months after HSRT. The intracranial objective response rate (ORR) and disease control rate (DCR) were evaluated via institutionally modified RECIST v1.1, and the minimum target brain lesion size was 5 mm in longest diameter. Irradiated lesion progression-free survival (il-PFS) was calculated from the day of radiotherapy initiation to the day of irradiated lesion progression, death, or the last day of follow-up. The intracranial PFS (iPFS) was calculated from the day of radiotherapy initiation to the day of intracranial disease progression, death, or the last day of follow-up. Given that patients with different cancer types were enrolled, the heterogeneity of systemic treatment and overall survival times were not included in the analysis. Treatment-related adverse events (TRAEs) were determined by the Common Terminology Criteria for Adverse Events v5.0. Cerebral radiation necrosis (CRN) was diagnosed on the basis of the following criteria: increased T1 contrast enhancement located in the irradiated area with increased peripheral edema on MRI and regression or stability of enhancing areas on serial follow-up MRI without additional treatment (20).

### 2.6 Statistics

Quantitative variables are described as medians (ranges), means [standard deviations (SDs)], and interquartile ranges (IQRs). Qualitative variables were described by their respective distribution modalities. The Kaplan−Meier method was used to visualize survival curves.

## 3 Results

Between March 2019 and February 2023, 68 patients were enrolled. The patients’ characteristics are shown in **Table 1**. Among patients unsuitable for surgical resection and one-fraction SRS, 51.3% (20/39), 25.6% (10/39), 15.4% (6/39), and 7.7% (3/39) had tumors located in or near the brainstem, optic nerves, cochlea, and capsula interna, respectively.

**Table 1:** Patient and tumor/treatment characteristics.

Characteristics	No./median (range)	%
Sex		
Male	35	51.5
Female	33	48.5
Age(y)	60.50 (29–84)	
<65	40	58.82
≥65	28	41.18
The reason for no surgical resection and SRS		
Unsuitable	39	57.4
Refused	29	42.6
Primary site of disease		
lung	48	70.59
breast	10	14.70
melanoma	8	11.76
Renal cell	2	2.95
Tumors		
GTV volume (cm^3^)	0.37 (0.06–1.26)	
≤0.5 cm^3^	45	66.67
0.5–1 cm^3^	23	33.33
PTV volume (cm^3^)	2.39 (0.49–4.00)	
≤2 cm^3^	24	35.29
2–3 cm^3^	26	38.24
3–4 cm^3^	18	26.47
Prescribed dose	30 (24–39)	
24Gy/3f	3	4.41
27Gy/3f	4	5.88
30Gy/3f	57	83.82
36Gy/3f	3	4.41
39Gy/3f	1	1.48
Systemic treatment		
TKIs	37	54.4
Chemotherapy	39	57.4
Immunotherapy	11	16.2

SRS, stereotactic radiosurgery; GTV, gross tumor volume; PTV, planning target volume.

**Figure 1:**
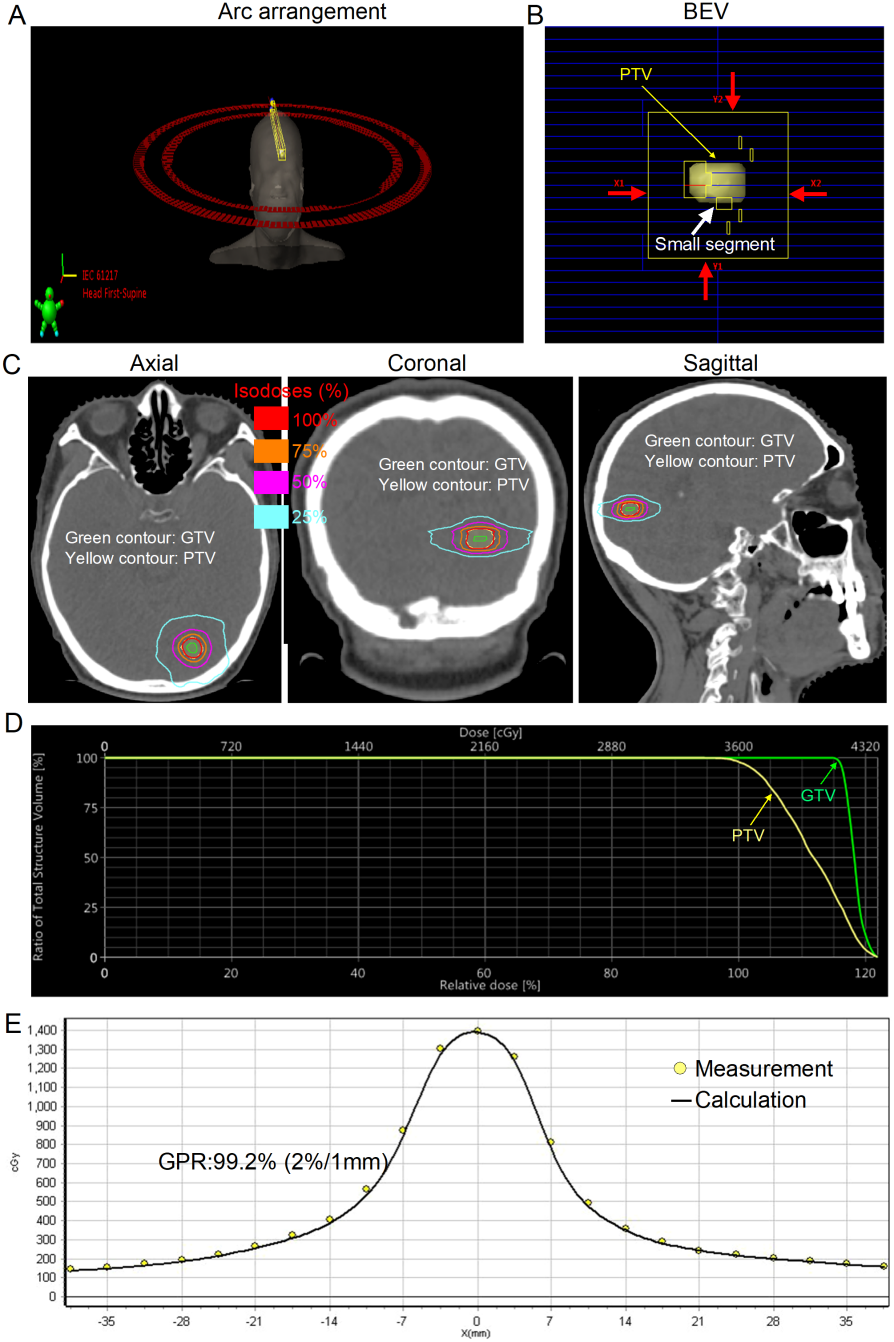
Typical arc arrangement and dosimetric results for a representative patient with small brain metastasis [gross tumor volume (GTV), 0.2 cm^3^; planning target volume (PTV), 0.7 cm^3^]. **(A)** Coplanar volumetric modulated arc therapy was used in this case (181°–179° and 179°–181°). **(B)** Beam’s eye view (BEV) after planning optimization with a jaw size of 3 cm×3 cm (red arrows). **(C)** Final spatial dose distribution. The green contour denotes the GTV. The yellow contour denotes the PTV. **(D)** Dose−volume histogram (DVH) for the PTV and GTV. **(E)** Dose comparison between the measured and calculated values along the X-axis. The measured and calculated dose values agree very well.

C-VMAT yielded clinically satisfactory treatment planning (**Table 2**). The median target coverage for all plans was 98% (range, 95–99.9%). The median CI, GI, and HI were 1.05 (range, 0.96–1.34), 4.30 (2.90–6.09), and 1.28 (range, 1.08–1.49), respectively. The median D_98%_ of the GTV was 34.82 Gy (range, 25.01–50.87 Gy). With respect to NBT, the median V_18Gy_, V_21Gy_, V_23Gy_, and D_mean_ were 7.29 cm^3^ (range, 1.40–13.31 cm^3^), 5.33 cm^3^ (range, 0.88–9.80 cm^3^), 4.40 cm^3^ (range, 0.61–8.22 cm^3^), and 0.88 Gy (range, 0.35–1.57 Gy), respectively. In terms of delivery accuracy, high gamma passing rates (GPRs) were achieved with all GPRs >90%, regardless of the criteria. The median MUs and BOT were 2,284.50 (range, 2,501.00–3,784.00) and 1.76 min (range, 1.13–2.91 min), respectively. The typical field arrangements and visual comparison of dose distributions in axial, coronal, and sagittal sections and the measured results of a representative patient with a PTV of 0.7 cm^3^ are shown in **Figure 1**.

**Table 2:** Various dosimetric parameter results.

Index	Dosimetry Variable	Mean (SD)	Median (Range)	IQR
Plan quality	Normalization	96.99 (1.79)	98.00 (95.00–99.90)	95.00–99.00
	GI	4.41 (0.60)	4.30 (2.90–6.09)	3.97–4.80
	CI	1.08 (0.10)	1.05 (0.96–1.34)	1.00–1.15
	HI	1.29 (0.09)	1.28 (1.08–1.49)	1.22–1.36
	D_98%_	35.11 (3.68)	34.82 (25.01–50.87)	33.56–36.39
NBT sparing	V_18Gy_	7.37 (2.34)	7.29 (1.40–13.31)	5.61–8.91
	V_21Gy_	5.44 (1.77)	5.33 (0.88–9.80)	4.12–6.66
	V_23Gy_	4.50 (1.51)	4.40 (0.61–8.22)	3.42–5.55
	D_mean_	0.89 (0.29)	0.88 (0.35–1.57)	0.65–1.03
Delivery efficiency	MUs	2,286.71 (499.55)	2,284.50 (2,501.00–3,784.00)	3,356.00–3,639.25
	BOT	1.76 (0.38)	1.76 (1.13–2.91)	1.47–1.94
Delivery accuracy	3%/1 mm (%)	99.58 (0.86)	100 (95.20–100)	99.60–100
	2%/2 mm (%)	99.60 (0.74)	100 (95.60–100)	99.38–100
	2%/1 mm (%)	99.02 (1.46)	99.65 (92.10–100)	98.60–100

SD, standard deviation; IQR, interquartile range; GI, gradient index; CI, target conformity index; HI, homogeneity index; NBT, normal brain tissue; D_98%_, minimal dose received by 98% of gross tumor volume; V_xGy_, volume of NBT receiving ≥x Gy; D_mean_, mean dose to NBT; MUs, monitor units; BOT, beam on time.

**Figure 2:**
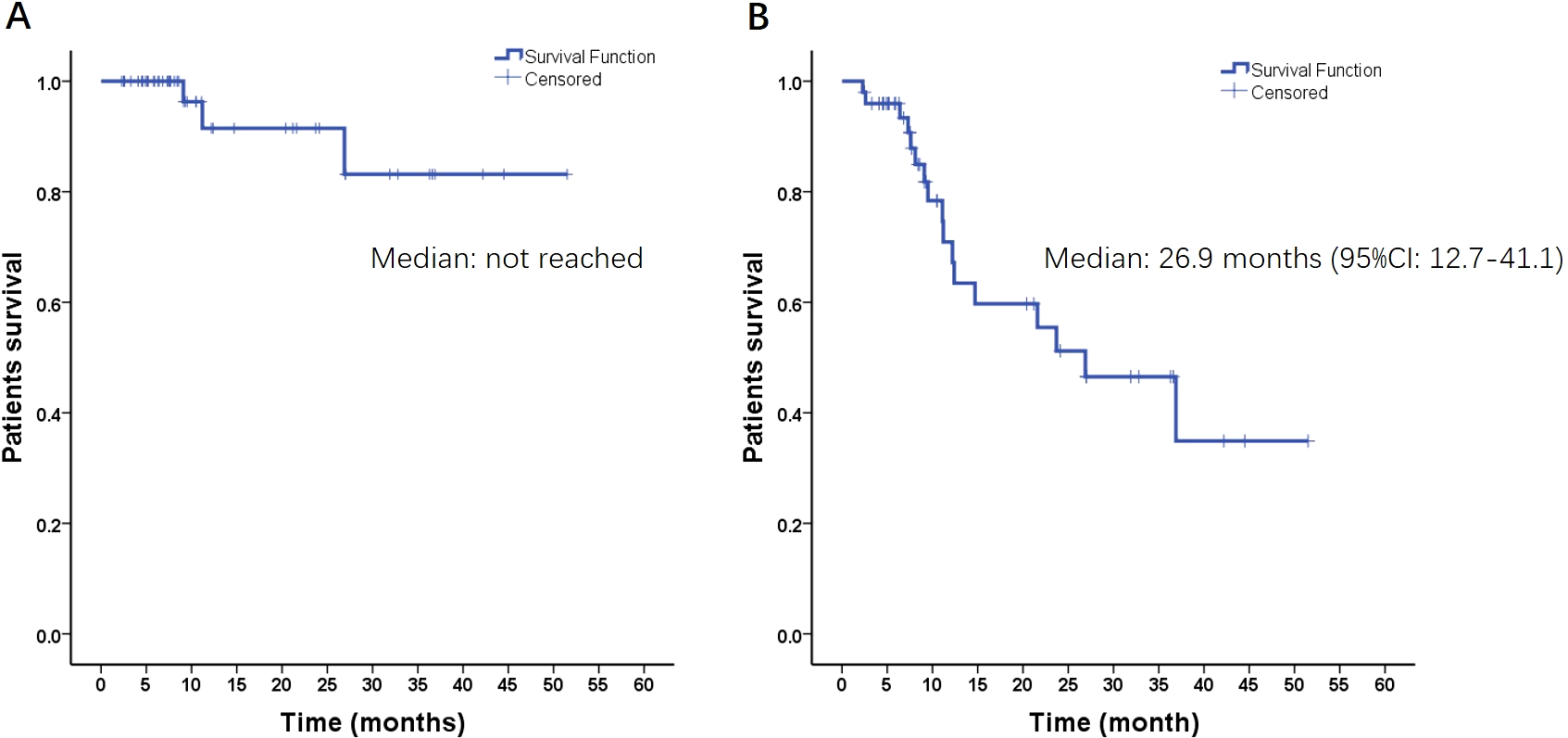
Radiation-induced lesion progression-free survival **(A)** and intracranial progression-free survival **(B)** of all patients.

As of June 2023, the median follow-up time was 9.1 months (range, 2.3–51.5 months). The intracranial ORR and DCR for all patients were 64% (95% CI, 57.3%–71.6%) and 96% (95% CI, 87.7%–99.4%), respectively. Only four irradiated lesions progressed at the end of follow-up, and the median IL-PFS was not achieved (**Figure 2A**). The median iPFS was 26.9 (95% CI, 12.7–41.1) months (**Figure 2B**). The 1-year and 2-year intracranial control rates were 70.9% (95% CI, 55.2%–86.6%) and 51.2% (95% CI, 32.6%–69.8%), respectively. The 1-year and 2-year LC rates were 91.5% (95% CI, 80.1%–100%) and 83.2% (95% CI, 64.6%–100%), respectively. Among the 23 patients who experienced intracranial relapse, 82% had new BMs, and 18% had both irradiated lesion progression and new BMs. Seven patients (10.3%) experienced grade 1–2 dizziness, five patients (7.4%) experienced CRN with a median duration of 12 (9–24) months after the end of HSRT, and no other TRAEs were observed.

## 4 Discussion

For large BMs, the treatment mode has shifted from SRS to HSRT, with satisfactory LC and limited CRN reported (21). In addition, the present retrospective, single-institution series indicated that Linac-based HSRT achieved favorable clinical efficacy with acceptable toxicity and satisfactory dosimetric parameters. To the best of our knowledge, the present study is the first report concerning the detailed dosimetric parameters and clinical outcomes of Linac-based C-VMAT 3F-HSRT with a 3-mm PTV margin for patients with small BMs who were unsuitable for or refused surgical resection and one-fraction SRS.

HSRT, similar to SRS, involves high doses per fraction, where a steep dose fall-off is extremely important to reduce the dose to surrounding NBTs or OARs. For single-target SRS, Torizuka et al. (22) showed that the addition of a non-coplanar arc in the VMAT plan can achieve better NBT sparing than can the addition of only coplanar arcs. However, non-coplanar delivery techniques may increase overall treatment time (OTT) and setup errors, especially for conventional non-invasive thermoplastic mask immobilization, as demonstrated by our previous studies (23, 24). In contrast, C-VMAT eliminates the setup errors derived from couch rotation and the need for radiotherapy staff to enter the treatment room for each couch rotation. More importantly, each BM was small in the present study, with a median PTV of 2.39 cm^3^ (range, 0.49–4.00 cm^3^), which was usually approximately a sphere and corresponded to a median diameter of 1.66 cm (range, 0.98–1.97 cm). Therefore, the C-VMAT plan was sufficient to achieve a clinically satisfactory treatment plan (**Table 2**; **Figure 1**). With only coplanar arcs, even when the patient’s setup (approximately 4 min) and CBCT verification time (approximately 2 min) were taken into consideration, each patient could be treated within 10 min in the present study, which could decrease the risk of intrafraction movement, as suggested by Fung et al. (25). For small BMs, there are also other challenges associated with the VMAT delivery technique, such as the dose calculation uncertainty associated with small MLC segments (11) (**Figure 1B**; white arrow). Interestingly, all the plans assured a GPR≥90% (GPR≥90% with the 3%/1mm evaluation criterion is our minimum value for the clinical usage of HSRT) for all the metrics. The higher GPR may be explained by the use of a fixed jaw size (3 cm×3 cm) (**Figure 1B**; red arrows), which has been shown to significantly improve the GPR (26).

CRN represents the most common late toxicity after HSRT. Several investigators have demonstrated the link between the specific isodose of NBT and the risk of CRN for BMs treated with HSRT. For 3F-HSRT, Minniti et al. (4, 27) reported that the 1-year risk of CRN was up to 24% and 14% for V_18Gy_>30.2 cm^3^ and V_21Gy_>20.9 cm^3^, respectively. In addition, another study revealed that for BMs treated with 3F-HSRT, a V_23Gy_≥7 cm^3^ was associated with a 10% risk of CRN (28). In the present study, most volumes of V_23Gy_, V_21Gy_, and V_18Gy_ were far below the abovementioned dose−volume threshold, and only 7.4% CRN was observed. From a dosimetric standpoint, the GTV-to-PTV margin should be extremely tight, thus reducing the V_18Gy,_ V_21Gy_, and V_23Gy_ volumes as much as possible. In the present study, a 3-mm PTV margin was used, which is significantly larger than most reported PTV expansions (typically 1–2 mm) (29–31). However, even with a 3-mm PTV margin, the irradiated volume was still limited because of the small GTV. This might be the reason for the limited V_18Gy,_ V_21Gy_, and V_23Gy_ volumes in the present study.

In previous reports, the 1-year intracranial LC rates were 85%–97% for patients with small BMs (<4 cm^3^ in volume or ≤2 cm in diameter) who received SRS (32–34). Similarly, in the present study, satisfactory LC was achieved, with 1-year and 2-year LC rates of 91.5% and 83.2%, respectively, and only four irradiated lesions (5.9%) progressed at the end of follow-up. Furthermore, new BM was the main intracranial relapse pattern, with an incidence rate of 33.8%, which might be caused by the inadequate intracranial efficacy of systemic treatment.

For HSRT, Dupic et al. (35) reported that the GTV D_98%_ was a significant predictive factor for LC, with 1-year LC rates of 91.9% and 69.6% for D_98%_≥29 Gy and <29 Gy, respectively. Lucia et al. (36) explored the impact of HI on LC in BM patients treated with 3F-HSRT and reported that an inhomogeneous target dose yielded a higher 1-year LC rate than did a homogeneous target dose (93% vs. 78%, *p*=0.005). In the present study, the median D_98%_ of the GTV was 34.82 Gy (IQR, 33.56–36.39 Gy), and the mean HI value was 1.29 (SD, 0.09), which means that inhomogeneous dose distributions were generated for most plans. These factors might partly explain the high intracranial LC rate achieved.

However, several limitations should be considered in this study, including its retrospective clinical design, heterogeneity of patient characteristics, and the fact that it only represents patients treated at a single institution, which may need to be confirmed by further prospective and multi-institutional investigations. Furthermore, the heterogeneity of systemic treatments could also affect the tumor response or survival of these patients. Nevertheless, we suggest that our study provides useful information, as it provides new insights into the role of Linac-based C-VMAT 3F-HSRT using a 3-mm PTV margin in treating patients with small BMs.

## 5 Conclusion

The use of C-VMAT in conjunction with a 3-mm PTV margin for 3F-HSRT in the treatment of small BMs provides excellent dosimetric results regarding target dose coverage, NBT sparing, delivery efficiency, and accuracy and yields excellent outcomes with acceptable toxicity. These clinical results encourage the implementation of 3F-HSRT for patients with small BMs who are unsuitable for or refuse surgical resection and SRS. In addition, further studies are needed to establish the optimal dose fractionation protocol for individual patients.

## Data Availability

The raw data supporting the conclusions of this article will be made available by the authors, without undue reservation.
